# A chip-scale atomic beam for nonclassical light

**DOI:** 10.1126/sciadv.aec3179

**Published:** 2026-06-03

**Authors:** Braden J. Larsen, Hagan Hensley, Gabriela D. Martinez, Alexander Staron, William R. McGehee, John Kitching, James K. Thompson

**Affiliations:** ^1^ JILA, University of Colorado, Boulder, CO, USA.; ^2^ National Institute of Standards and Technology, Boulder, CO, USA.; ^3^ Department of Physics, University of Colorado, Boulder, CO, USA.

## Abstract

Nonclassical light is a critical resource for a broad range of quantum technologies and fundamental science. However, leading platforms such as laser-cooled atoms and solid-state systems both present substantial challenges to scalability due to complexity and spectral drift. Here, we demonstrate a hybrid approach using a chip-scale rubidium beam coupled to a high-finesse cavity–quantum electrodynamics (QED) system. Specifically, we generate nonclassical light and observe optical nonlinearities at the few photon level. This is achieved without degradation of the cavity-QED system. By demonstrating the compatibility of these two technologies, we open a path for distributed sources of nonclassical light and set the stage for using cavity-QED to enhance the performance of chip-scale magnetometers and atomic clocks.

## Teaser

Combining a chip-scale atomic source with an optical cavity, the nonlinearity of an atom is used to produce nonclassical light.

## INTRODUCTION

Rapid progress is being made across multiple disciplines to generate nonclassical light for a broad range of scientific applications, including quantum networks (*1*), computing (*2*), metrology (*3*), and tests of quantum foundations (*4*). Integrated solid-state systems have made progress (*5*, *6*) but can suffer from spectral drift (*7*) of the photons that they emit and require cryogenic environments. Laser-cooled and trapped atoms (*8*–*10*) sidestep spectral drift but at the cost of a high degree of complexity, with optical and vacuum systems covering multiple optical tables. The complexity of both platforms remains an open challenge for scaling these systems for scientific and technological applications.

In parallel, there has been renewed recent interest in examining whether room-temperature atomic vapors or fast atomic beams can be used as a platform for cavity–quantum electrodynamics (cavity-QED). Using hot atoms has shown the ability to provide technological and scientific solutions, including phase coherent atomic driving (*11*), mapping the vacuum state of the cavity mode (*12*), and performing optical magnetometry (*13*). To enhance scalability, many groups are pursuing integrated chip-scale devices that interact with atomic vapors (*14*–*17*) with applications such as electrometry (*18*), magnetometry (*19*, *20*), inertial sensing (*21*), and atomic clocks (*22*, *23*). A recent innovation has been the development of a fully enclosed and compact Rb beam source, whose collimation is achieved using etched micrometer-sized channels (*24*). This type of source was used recently to realize a chip-scale microwave atomic beam clock using Ramsey coherent population trapping (*25*).

Here, we demonstrate a hybrid approach combining a chip-scale atomic beam source and a freestanding optical cavity. Specifically, we demonstrate the first capabilities of such a hybrid system by generating nonclassical light and optical nonlinearities at the few photon level. This approach offers a scientifically enabling high data rate, here emphasized by the fact that we can observe both a nonclassical second-order and third-order Glauber coherence. We further exploit the third-order coherence to realize a heralded sub-Poissonian light source. We observe that the optical cavity is not degraded when the atomic beam is introduced, avoiding a challenging problem when coupling thermal vapors to nanophotonic optical devices. Furthermore, the success of this hybrid approach will also provide a path to enhance other atomic-based quantum science and technologies, such as clocks, field sensing, and inertial sensing, by greatly enhancing state preparation, manipulation, and measurement. Although our device is not yet a fully chip-scale vacuum system, innovations in integrated mirror substrates (*26*) provide a path forward for even greater integration of this hybrid approach for reducing complexity and enhancing scalability.

## RESULTS

### Chip-scale atomic beam

**Fig. 1.**
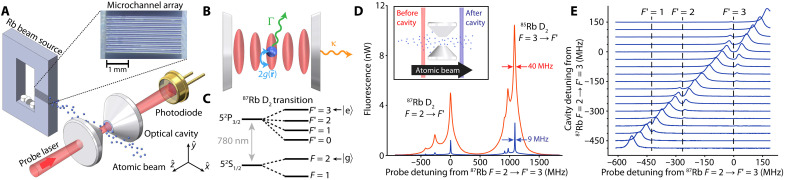
Experimental setup. (**A**) The compact atomic source (gray box) produces a thermal atomic beam (blue spheres) by feeding an atomic vapor of Rb through a microchannel array (inset), which propagates through an optical cavity formed by two high-finesse mirrors (white cones). Atoms are excited using a weak probe beam, while the photons transmitted through the cavity are collected using a photodiode (gold). (**B**) Diagram of the atom-cavity interactions and dissipation mechanisms. Rb87 atoms (blue spheres) in *F* = 2 are coupled to the cavity mode (red ovals) at the vacuum Rabi frequency 2g(r). Excitations in the atom-cavity system are dissipated through atomic spontaneous emission at rate Γ or cavity power decay at rate κ. (**C**) Rb87 $D_{2}$ transition hyperfine structure. (**D**) Fluorescence spectroscopy of the atomic beam perpendicular to its direction of propagation. The Rb87 and Rb85 $D_{2}$ transitions are characterized 10 mm before (red) and after (blue) the optical cavity. (**E**) The normal mode splitting of the atom-cavity system on the three allowed Rb87 F=2→F' hyperfine transitions.

The miniature atomic beam source is shown in Fig. 1A. The device is a modification of the chip-scale atomic beam devices that have been used to realize a miniature atomic beam clock (*24*, *25*), here adapted to provide an atomic beam through the center of an optical cavity. The beam source consists of a silicon wafer with an internal reservoir that contains a Rb vapor. A microchannel array is etched into the silicon surface and is fed by the vapor to produce a series of atomic beams along the axis of the microchannels. The device is enclosed by two layers of borosilicate glass. One glass layer is anodically bonded to the silicon to form a hermetic seal, while the other is mechanically affixed to the structure to allow for easy loading and reloading of alkali metal into the device. The beam source is placed inside an actively pumped vacuum chamber, and the atomic beams are aligned with the cavity opening.

The microchannel array consists of 10 rectangular channels spaced by 150 μm center to center. Each channel has a length of 3 mm and a square cross section of 100 μm by 100 μm. The channels’ aspect ratio (ratio of length to width) is 30, which creates atomic beams with a half-angle divergence of roughly 33 mrad (*27*). Rubidium is introduced into the beam device using Rb molybdate Zr/Al pill-type dispensers, which are thermally activated using laser heating to produce pure Rb metal (*28*), as described in Materials and Methods. Once the Rb is deposited, the beam device is resistively heated up to 180°C to control the Rb vapor pressure within the vapor cell region. For most of the work here, we operate at source temperatures between 40° and 70°C, where the total atomic flux from the array varies from $10^{10}$ to $2\times 10^{11}\;s^{-1}$. A single Rb pill contains 0.4 mg of natural abundance Rb, and this is expected to sustain source operation at 70°C for ~5 months. Future devices could incorporate 10 of these millimeter-scale pills for source operation lasting ~4 years at 70°C.

### Optical cavity

Our Fabry-Pérot microcavity is similar to those of (*29*–*34*). More advanced and integrated designs (*35*–*38*) could be considered for future work, but the goal in the present context is to understand whether low-transmission mirror coatings that are common to all of these other approaches are compatible with the miniature beam source at a level sufficient to produce nonclassical light fields.

For the results presented here, we used two different cavities that we will refer to as A and B. All mirrors used were either low or high transmission, with power transmission of $2\times 10^{-6}$ and $40\times 10^{-6}$, respectively, at the atomic transition wavelength $\lambda_{a}\approx 780.2$ nm. For cavity A, we use two low-transmission mirrors so that light exits the cavity equally from both mirrors. For cavity B, we use one low- and one high-transmission mirror so that most of the light exits via the high-transmission end. Both cavity lengths were set to L=80(5) μm, and their resonance frequencies were tuned near the Rb87 $D_{2}|5^{2}S_{1/2},F=2\rangle \to |5^{2}P_{3/2},F^{\prime }=3,2,1\rangle$ transitions (Fig. 1C), which have a decay rate of Γ≈2π×6.1 MHz (*39*). The fundamental transverse electromagnetic (TEM_00_) mode had a waist of $w_{0}=22.3\left(4\right)\;\text{μm}$ at $\lambda_{a}$. For cavities A and B, respectively, the measured cavity linewidths are κ/2π=33(1) and 13(1) MHz, indicating per mirror power loss coefficients of $15\left(3\right)\times 10^{-6}$ and $1\left(1\right)\times 10^{-6}$. Their corresponding finesses are $F_{A}=0.6\left(1\right)\times 10^{5}$ and $F_{B}=1.5\left(1\right)\times 10^{5}$.

The atom-cavity system operates in the strong-coupling limit as characterized by the single-particle cooperativity $C=4g_{0}^{2}/\kappa \Gamma \gg 1$, where $2g_{0}$ is the vacuum Rabi frequency describing the coherent atom-cavity interactions (*40*). In the limit $\kappa \gg 2g_{0},\Gamma$, the cooperativity characterizes the probability $P_{c}=C/\left(1+C\right)$ that an atom in the optically excited state decays to the ground state by emitting a photon into the cavity (*41*). Given the cavity effective mode volume $V=\pi w_{0}^{2}L/4$ and dipole moment for the Rb87 $D_{2}$ cycling transition $|F=2,m_{F}=2\rangle \to |F^{\prime }=3,m_{F^{\prime }}=3\rangle$, the vacuum Rabi frequency at an antinode on the cavity axis is $2g_{0}=2\pi \times 52\left(2\right)\;\text{MHz}$, yielding peak cooperativities of C=18(2) and 35(3) for cavities A and B, respectively. As the atoms transit the standing wave cavity mode, the coupling will vary with position as

$$g\left(r\right)=g_{0}\text{cos}\left(\frac{2\pi z}{\lambda_{a}}\right)e^{-\frac{x^{2}+y^{2}}{w_{0}^{2}}}$$ (1)

where *z* is the position along the cavity axis and *x* is the position along the atomic beam direction. To account for the inhomogeneous coupling of atoms within the standing wave profile of the cavity, we define an effective vacuum Rabi frequency $2g_{\text{eff}}=\sqrt{3/8}\times 2g_{0}=2\pi \times 32\left(2\right)\;\text{MHz}$ and a time-averaged effective atom number $N_{\text{eff}}$ for a two-level atom (see Supplementary Materials), in a fashion reminiscent to the effective couplings used in cavity-aided spin squeezing (*42*–*44*).

### Interfacing the atomic source with the cavity

The output of the Rb source is 10 mm from the cavity mode. The angular alignment of the atomic beam is precise to within a few milliradians, tuned using a dispersive feature in the cavity probe transmission (*45*), as described in the Supplementary Materials. The mean thermal velocity of v≈290 m/s at 70°C gives an average cavity transit time of $t_{T}\approx 150\;\text{ns}$ across the cavity mode diameter $2w_{0}$. The root mean square (RMS) angular divergence of the atomic beam is calculated from the linewidth of the Rb85 $F=3\to F^{\prime }=4$ transition as measured using fluorescence spectroscopy (Fig. 1D). Just before the cavity, the RMS angular divergence of the full atomic beam is 43(3) mrad. However, the portion of the beam that passes through the cavity has an RMS angular divergence of only 8(2) mrad such that the RMS displacement along the cavity axis $\hat{z}$ is only $0.23\lambda_{a}$ per mode waist $w_{0}$ traveled along $\hat{x}$.

### Demonstrating the atom-cavity coupling

As seen in Fig. 1E, the atom-cavity coupling hybridizes the atomic and cavity modes, leading to a series of avoided level crossings in the measured cavity-probe transmission of cavity A (Fig. 1A) versus probe frequency (*46*), with the cavity resonance frequency $\omega_{c}$ varied over a range spanning the Rb87 $|F=2\rangle \to |F^{\prime }=3,2,1\rangle$ transitions between sweeps. As the cavity comes into resonance with each transition, the single cavity resonance frequency splits into two normal modes.

**Fig. 2.**
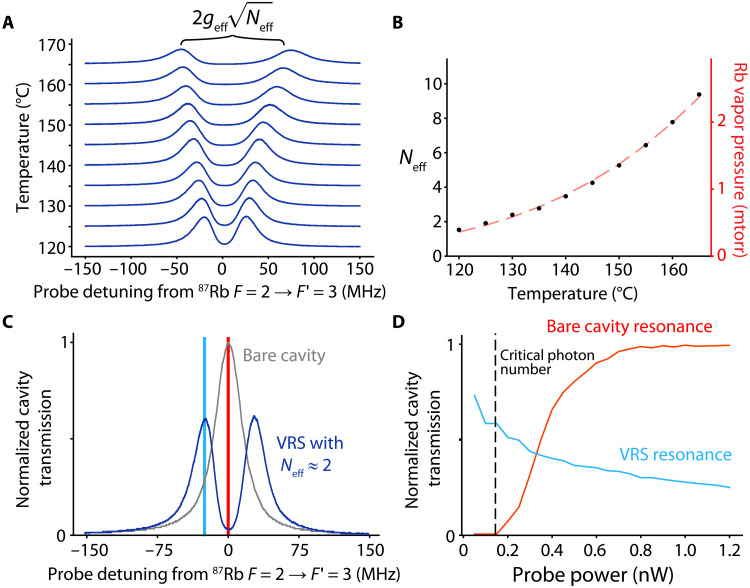
Demonstration of the cavity QED platform. (**A**) Splitting of the VRS peaks increases as a function of the source temperature. (**B**) The temperature dependence of $N_{\text{eff}}$ (black dots) agrees with the Rb vapor pressure curve (red dashed line) up to a multiplicative scaling factor, consistent with the expectation that the VRS scales with $\sqrt{N_{\text{eff}}}$. (**C**) The transmission in (C) and (D) is normalized to the peak transmission of the bare cavity. Bare cavity transmission as a function of probe detuning is shown in gray. Transmission with $N_{\text{eff}}\approx 2$ is shown in blue, showing VRS peaks. (**D**) Nonlinear cavity transmission as a function of the probe power. The blue and red curves are taken at detunings corresponding to the blue and red lines in (C). These data were also taken with $N_{\text{eff}}\approx 2$.

In Fig. 2A, we observe the hybridized modes (sometimes called polaritons) when we set the cavity to resonance with the $|F=2\rangle \to |F^{\prime }=3\rangle$ transition $\omega_{a}$. $N_{\text{eff}}$ and $g_{\text{eff}}$ are defined so that the frequency splitting or vacuum Rabi splitting (VRS) of the two hybridized modes is $2g_{\text{eff}}\sqrt{N_{\text{eff}}}$. We vary the Rb source temperature and measure the splitting to determine $N_{\text{eff}}$ as a function of temperature. The scaling of $N_{\text{eff}}$ matches the behavior of the known Rb vapor pressure curve (*47*) up to a constant scale factor (Fig. 2B). We can also resolve the separation of the VRS peaks for $N_{\text{eff}}\approx 1$. The full width at half maximum (FWHM) linewidth of each hybridized normal mode is predicted to be (κ+Γ)/2=2π×19.5(5) MHz compared to the measured linewidth 22(1) MHz, which we attribute to additional broadening from Poisson variance in the atom number.

Unlike the cavity mode, each atom can carry only one optical excitation. This is reflected in the nonlinear transmitted power versus probe power beginning at an intracavity photon number of roughly 3(1) (Fig. 2, C and D), known as the critical photon number $n_{0}$ (*40*, *48*, *49*). This few-photon optical nonlinearity could be used for optical switching at the few photon level.

### Second-order coherence

**Fig. 3.**
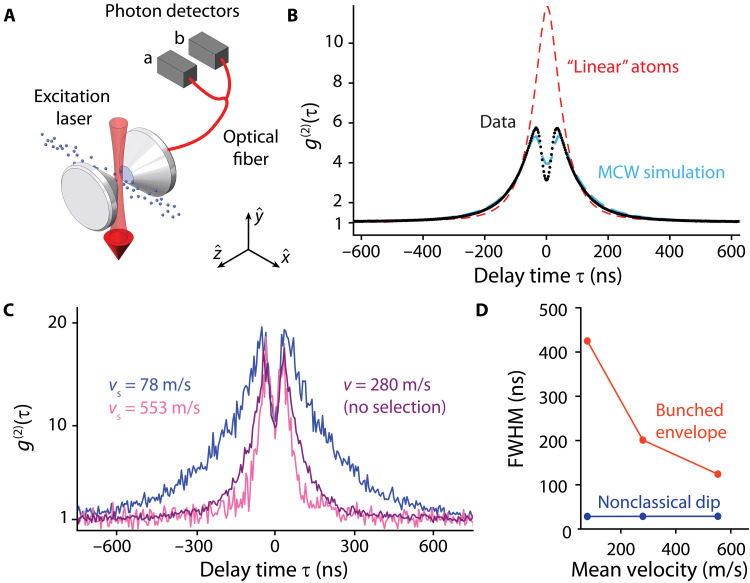
Nonclassical light. (**A**) An excitation beam overlaps with the cavity mode to continuously excite atoms during their transits. The cavity output is collected on two single-photon counting modules (SPCMs). (**B**) Measured profile of $g^{\left(2\right)}\left(\tau \right)$ (black dots). The $g^{\left(2\right)}\left(\tau \right)$ displays a bunched profile and a nonclassical dip within the bunched envelope that is centered at τ=0. The dashed blue line is a Monte Carlo wave function (MCWF) simulation; the details of which are explained in the Supplementary Materials. The dashed red line is a classical model for the bunched envelope, ignoring the nonlinearity of the atoms. (**C**) Velocity-selective pumping. Relying on the Doppler shift, we use optical pumping to pump atoms into *F* = 2 with a desired velocity. Profiles of $g^{\left(2\right)}\left(\tau \right)$ are shown with no optical pumping (purple), $v_{s}\approx 78\;m/s$ (blue), and $v_{s}\approx 553\;m/s$ (pink). (**D**) The width of the bunched envelope changes to reflect the transit time, while the width of the nonclassical dip is constant to within measurement uncertainty.

The nonlinearity of the atoms is next used to realize a nonclassical light source. We reduce the Rb source temperature to lower the effective atom number to $N_{\text{eff}}\approx 0.1$, limiting the likelihood of two atoms transiting the cavity mode at the same time. Cavity B’s resonance frequency is tuned to resonance with $\omega_{a}$. The atoms are now directly excited at frequency $\omega_{a}$ using a laser beam along $-\hat{y}$ (see Fig. 3A) with a Rabi frequency of $\Omega =2\pi \times 36_{-18}^{+5}\;\text{MHz}$.

Photons exiting the cavity are sent to a nonpolarizing 50/50 beamsplitter, and each output is directed to a single-photon counting module (SPCM). The detected photons’ statistics are characterized using the second-order correlation function $g^{\left(2\right)}\left(\tau \right)$ (*50*). Given the detection of a photon at time *t*, $g^{\left(2\right)}\left(\tau \right)$ gives the relative likelihood to detect a second photon at time t+τ compared to what we would expect if the arrival times of photons were uncorrelated or Poissonian. $g^{\left(2\right)}\left(\tau \right)>1$ indicates that a second photon is more likely to arrive than for Poissonian light, whereas $g^{\left(2\right)}\left(\tau \right)<1$ indicates that a second photon is less likely to arrive than for Poissonian light.

The measured $g^{\left(2\right)}\left(\tau \right)$ in Fig. 3B features a bunched envelope that asymptotes to 1 at large τ. Within this envelope, a central feature appears as a dip with an FWHM of $t_{\text{dip}}=28\left(3\right)\;\text{ns}$ and a local minimum $g^{\left(2\right)}\left(0\right)=3.13\left(1\right)$. This feature violates the classical inequality $g^{\left(2\right)}\left(0\right)>g^{\left(2\right)}\left(\tau \right)$, certifying that the light source is nonclassical (*51*). The dip conceptually arises from the time scale for the atom to return to the optically excited state after it has emitted a photon. The minimum value of $g^{\left(2\right)}\left(0\right)$ is limited by the probability of multiple photons being deposited in the cavity at the same time. This can happen because of either the finite storage time 1/κ of the cavity or because of multiple atoms transiting the cavity at once.

The characteristic timescale for the bunching envelope is that of the atom number fluctuations or the average atom transit time $t_{T}\approx 150\;\text{ns}$ (*51*). The red dashed curve of Fig. 3B shows the expected $g^{\left(2\right)}\left(\tau \right)$ if the atoms were classical linear scatterers transiting the cavity with random atom arrival times, following the thermal atomic velocity distribution and the measured angular beam divergence along the cavity axis. This model generally reproduces the shape of the bunched envelope but does not display a central dip.

Modeling the full shape requires capturing the nonlinearity of the atoms, which we do using a Monte Carlo wave function (MCWF) simulation (the dashed blue line in Fig. 3B), as described in the Supplementary Materials. The values of $g_{0}$ and Ω are adjusted to better describe the observed dip. We also observe how varying the cavity linewidth and atomic beam flux affects $g^{\left(2\right)}\left(\tau \right)$. These simulations support the physical intuition that the finite cavity-linewidth κ is the primary limitation on the minimum value of $g^{\left(2\right)}\left(0\right)$ because the associated storage time 1/κ blurs the time dynamics.

We can vary the interaction time with the cavity using velocity-selective optical pumping of atoms from the ground state *F* = 1 (which does not resonantly interact with the cavity) to *F* = 2 (which does resonantly interact with the cavity). In Fig. 3C, we show the measured $g^{\left(2\right)}\left(\tau \right)$ with no selection and for $v_{s}\approx 78$ and 553 m/s. The observed bunching timescale changes by ≈2 without changing the width of the nonclassical central feature, as shown in Fig. 3D.

### Third-order coherence

**Fig. 4.**
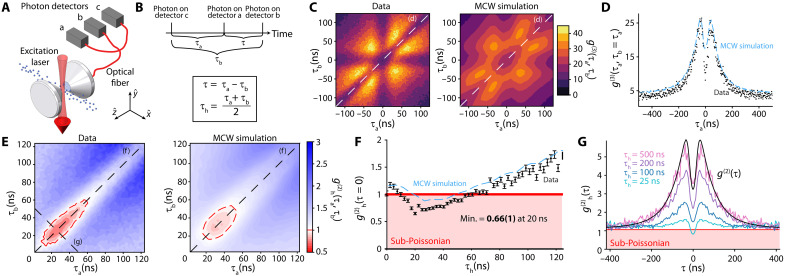
$g^{\left(3\right)}\left(\tau_{a},\tau_{b}\right)$ and heralded $g^{\left(2\right)}\left(\tau \right)$ measurement. (**A**) To measure $g^{\left(3\right)}\left(\tau_{a},\tau_{b}\right)$ and heralded $g^{\left(2\right)}\left(\tau \right)$, we add a third SPCM. (**B**) To compare $g_{h}^{\left(2\right)}\left(\tau_{a},\tau_{b}\right)$ to nonheralded $g^{\left(2\right)}\left(\tau \right)$, we redefine our variables: $\tau_{h}$ is the wait time after the herald photon, and τ is the time difference between the two heralded photons. (**C**) Three-photon correlation $g^{\left(3\right)}\left(\tau_{a},\tau_{b}\right)$: data (left) and theory (right). It can be seen that $g^{\left(3\right)}\left(0,0\right)<g^{\left(3\right)}\left(\tau_{a},\tau_{b}\right)$ for some nonzero values of $\tau_{a},\tau_{b}$, which is also an indicator of nonclassical light. (**D**) A profile along the diagonal of (C) with $\tau_{a}=\tau_{b}$ again shows $g^{\left(3\right)}\left(0,0\right)<g^{\left(3\right)}\left(\tau_{a},\tau_{b}\right)$. The blue dashed curve shows the same profile for the theory. (**E**) A full two-dimensional plot of $g_{h}^{\left(2\right)}\left(\tau_{a},\tau_{b}\right)$: data (left) and theory (right). The red region along the diagonal has $g_{h}^{\left(2\right)}\left(\tau_{a},\tau_{b}\right)<1$. (**F**) A profile along the diagonal of (E) can be interpreted as showing $g^{\left(2\right)}\left(0\right)$ as a function of the wait time $\tau_{h}$ after the herald photon. $g^{\left(2\right)}\left(0\right)$ drops below 1 for roughly $\tau_{r}<\tau_{h}<t_{T}$. The blue dashed curve shows the same profile for the theory. (**G**) As an alternate perspective, profiles of $g_{h}^{\left(2\right)}\left(\tau \right)$ along lines of constant $\tau_{h}$ drop below 1 for small $\tau_{h}$ but approach the nonheralded $g^{\left(2\right)}\left(\tau \right)$ in the limit of $\tau_{h}\gg t_{T}$. In this limit, the postselection is on random time windows rather than specifically times when there is an atom in the cavity, so we recover the nonheralded bunching behavior.

The thermal atomic beam source affords a high data rate of $\approx 10^{5}$ photon counts/s at 65°C, which is integrated for 8 hours to produce the data shown in Figs. 3 and 4. This is sufficient to characterize the photons’ third-order correlation, $g^{\left(3\right)}\left(\tau_{a},\tau_{b}\right)$ (*50*) (see Fig. 4, A and B) using three SPCMs labeled a, b, and c to avoid detector dead time. The measured $g^{\left(3\right)}\left(\tau_{a},\tau_{b}\right)$ shown in Fig. 4C also displays bunching due to the random transit times of the atoms through the cavity. The sixfold symmetry corresponds to the six permutations of labels for the three detected photons. Classical light obeys the relation $g^{\left(3\right)}\left(\tau_{a},\tau_{b}\right)\leq g^{\left(3\right)}\left(0,0\right)$, which our measured $g^{\left(3\right)}\left(\tau_{a},\tau_{b}\right)$ violates as seen in Fig. 4C and as emphasized by a cut along the white dashed line $\tau_{a}=\tau_{b}$ shown in Fig. 4D. For comparison, the results in Fig. 4 are in good agreement with the corresponding correlation functions computed using the same MCWF simulations from the previous section.

### Heralded second-order coherence

With a thermal atomic beam, we do not have the degree of control over the atoms that would be needed to suppress fluctuations in atom number. However, because we are not probing along the cavity axis, the detection of a photon from the cavity acts as a signal that there is an atom in the cavity. Thus, we can postselect on the presence of an atom in the cavity by requiring the detection of an initial cavity photon. In practice, this allows us to use the $g^{\left(3\right)}\left(\tau_{a},\tau_{b}\right)$ data to form a heralded second-order correlation measurement (*52*, *53*) shown in Fig. 4E that is sub-Poissonian $g_{h}^{\left(2\right)}\left(\tau_{a},\tau_{b}\right)<1$ within the dashed red region. Explicitly, the heralded correlation function is calculated as

$$g_{h}^{\left(2\right)}\left(\tau_{a},\tau_{b}\right)=\frac{M_{\text{cab}}\left(\tau_{a},\tau_{b}\right)M_{c}}{M_{\text{ca}}\left(\tau_{a}\right)M_{\text{cb}}\left(\tau_{b}\right)}$$ (2)

Here, detector c is taken to be herald detector, and correlations are calculated between the other two detectors a and b conditioned on the prior arrival of a photon in c. The heralded second-order correlation is a function of two times, $\tau_{a}$ being the time difference between photons c and a and $\tau_{b}$ between photons c and b (Fig. 4B). Each *M* represents the number of single ($M_{c}$), double ($M_{\text{ca}},M_{\text{cb}}$), or triple photon detections ($M_{\text{cab}}$), with double or triple coincidences occurring on different detectors at time differences $\tau_{a}$ and $\tau_{b}$.

The diagonal cut shown in Fig. 4F is equivalent to a heralded second order correlation function at $\tau \equiv \tau_{a}-\tau_{b}=0$. The minimum observed $g_{h}^{\left(2\right)}\left(0\right)$ is 0.66(1) at $\tau_{h}=20\;\text{ns}$, confirming that we have sub-Poissonian light. Similarly to $g^{\left(2\right)}\left(0\right)$, the minimum value of $g_{h}^{\left(2\right)}\left(0\right)$ is also limited by the finite decay time of the cavity, which allows for multiple excitations to be deposited within the cavity mode. The minimum occurs at $\tau_{h}\neq 0$ because the atom requires a finite reset time after having emitted the herald photon. At larger times $\tau_{h}$, the atom has left the cavity such that the heralding no longer applies and one returns to a bunching signal once again.

The opposite diagonal cuts shown in Fig. 4G at fixed herald times $\tau_{h}$ is equivalent to a $g_{h}^{\left(2\right)}\left(\tau \right)$. One sees that the light is antibunched at τ=0 for $\tau_{h}=25\;\text{ns}$ with reduced bunching away from τ=0. The antibunching disappears at larger heralding times $\tau_{h}$, returning to the previous unheralded result (black) at $\tau_{h}=500\;\text{ns}\gg t_{T}$.

## DISCUSSION

These results demonstrate that the miniature and mass-producible rubidium beam source is compatible with achieving C≫1 without degradation of the optical cavity over time due to Rb adhering to the mirror surface. With the source active between 40° and 70°C for 6 months, we observed no discernible change in the linewidth of the cavity at the level of ±10%, constraining the increase in losses to $\pm 0.5\times 10^{-6}$ per mirror/month. This would be sufficient to operate a cavity with a finesse of $10^{5}$ for 3 years without being loss dominated.

Ultimately, this experiment serves as a first step toward creating a miniaturized cavity QED system with a thermal atomic beam. This hybrid approach demonstrates a path for enhanced scalability of distributed sources of nonclassical light. While our atomic source is chip scale, our optical cavity is freestanding and enclosed within a larger vacuum chamber. We are currently exploring the integration of these cavities into sealed and passively vacuum pumped chip-scale devices such as in previously successful work (*25*). We plan to anodically bond the cavity mirrors within the chip-scale vacuum cell and use a two-stage channel design [as in (*24*)] to minimize getter outgassing and Rb vapor around the cavity. This device will find broad applications in miniature atomic clocks, chip-scale magnetometers, low-power optical switching, and generation of nonclassical light.

## MATERIALS AND METHODS

### Details of mirror machining

The concave mirrors with curvature *R*_c_ = 10 cm and diameter of *D*_m_ = 7.75 mm geometrically limit the on-axis mirror separation *L* to be $L\geq D_{m}^{2}/4R_{c}=150\;\mathrm{\mu m}$ so that the mirrors do not touch at their edges, which would leave no gap for atoms to enter the cavity. To achieve smaller mirror separations, the 4-mm-thick fused silica substrates were machined to form a cone starting from their base diameter of 7.75 mm down to a diameter of $D_{m}=1.5\;\text{mm}$ at the mirror surface. In principle, this allows for a minimum cavity length of L=6 μm at zero gap.

A computer numerical control machine is used to cone down each mirror to the desired diameter. First, the antireflective (AR)–coated side of the mirror is waxed onto a positioning fixture with PELCO Quickstick 135 Mounting Wax. The high-finesse surface is then sprayed with several layers of clear acrylic (Krylon Industrial, ACRYLI-QUIK) to protect it during machining. Starting 0.5 mm from the AR-coated surface, a medium grit diamond grinding wheel is used to cut the substrate at an angle of 41.8° from the normal of the mirror surface.

We perform 12 different cuts in four sequences to bring the substrate down to a cone with a diameter of 1.5 mm at the high-finesse surface. We check that the mirror is still waxed on the fixture between each cut. After machining, acetone dissolves the wax and acrylic from their respective surfaces.

### Depositing Rubidium in source region

Rubidium is deposited in the source region using a 4-W, 975-nm laser from QPhotonics to heat AlkaMax pills from SAES for 15 min. After the pills are activated, we use a resistive cartridge heater (Thorlabs, HT15W) to heat the entire device to temperatures between 40° and 180°C. This heating adjusts the vapor pressure of the rubidium trapped within the source, which determines the flux of rubidium leaving through the microchannel array. The mean thermal velocity of our atomic beam flux varies from ≈275 to 330 m/s over our operating temperature range. The pills can be activated up to 10 times using this procedure before they are depleted.

### Characterizing the divergence of the atomic beam

To characterize the angular divergence of the atomic beam before and after passing through the cavity, we perform fluorescence spectroscopy 10 mm before and after the optical cavity. Before the cavity, the Rb85 $F=3\to F^{\prime }=4$ transition has a measured linewidth of 40 MHz in agreement with (*25*). This is equivalent to an RMS angular divergence of 43(3) mrad. A similar measurement on the atoms exiting the cavity gives a much smaller linewidth of 9 MHz and an RMS angular divergence of 8(2) mrad. The narrow cavity acts as a spatial filter, selecting a population of atoms with less angular divergence to interact with the cavity mode.

### Cavity length stabilization

The cavity frequency is stabilized using a Pound-Drever-Hall (PDH) lock to a laser that is, in turn, locked at variable frequency offset from the Rb85 D_2_ *F* = 3 to *F* = 4 transition. The PDH laser light is sufficiently strong to saturate any atoms in the cavity, allowing us to measure the bare cavity-resonance frequency.

The PDH laser light is alternately toggled on and off for 220 μs, faster than the unity gain frequency of the cavity feedback loop. Science is done during the off period, during which the field emitted from the opposite end of the cavity is detected on 2 to 3 SPCMs. The SPCMs are gated off during the PDH probe period.

### Velocity-selective optical pumping

To verify that the characteristic timescale of the bunching corresponds to the average atom transit time, we use velocity-selective optical pumping to modify the shape of the peak. After optically pumping the population from ∣F=2〉 to ∣F=1〉, a beam angled $\theta =58{\left(1\right)}^{\circ }$ from the horizontal with detuning δ from the $|F=1\rangle \to |F^{\prime }=2\rangle$ transition is used to pump the atoms back into ∣F=2〉.

Because the velocity-selective pump has a component parallel to the atomic beam, we can select atoms moving at velocity $v_{s}$ by choosing $\delta =v_{s}\text{cos}\left(\theta \right)/\lambda_{a}$ to the red of the transition. The width of the velocity selection is broadened by excitations of the atomic beam by the vertical component of the optical drive and through velocity classes that are excited on the $|F=2\rangle \to |F^{\prime }=1\rangle$ transition.

## Supplementary Materials

### This PDF file includes:

Supplementary Text Fig. S1 References

## References

[1] M. Zou, Y. M. He, Y. Huang, J. Y. Zhao, B. C. Li, Y. P. Guo, X. Ding, M. C. Xu, R. Z. Liu, G. Y. Zou, Z. Ning, X. You, H. Wang, W. X. Pan, H. T. Zhu, M. Y. Zheng, X. P. Xie, D. Qin, X. Jiang, Y. H. Huo, Q. Zhang, C. Y. Lu, X. Ma, T. Y. Chen, J. W. Pan, Realization of an untrusted intermediate relay architecture using a quantum dot single-photon source. Nat. Phys. 21, 1670–1677 (2025).

[2] J. M. Arrazola, V. Bergholm, K. Brádler, T. R. Bromley, M. J. Collins, I. Dhand, A. Fumagalli, T. Gerrits, A. Goussev, L. G. Helt, J. Hundal, T. Isacsson, R. B. Israel, J. Izaac, S. Jahangiri, R. Janik, N. Killoran, S. P. Kumar, J. Lavoie, A. E. Lita, D. H. Mahler, M. Menotti, B. Morrison, S. W. Nam, L. Neuhaus, H. Y. Qi, N. Quesada, A. Repingon, K. K. Sabapathy, M. Schuld, D. Su, J. Swinarton, A. Száva, K. Tan, P. Tan, V. D. Vaidya, Z. Vernon, Z. Zabaneh, Y. Zhang, Quantum circuits with many photons on a programmable nanophotonic chip. Nature 591, 54–60 (2021). 33658692 10.1038/s41586-021-03202-1 PMC11008968

[3] G. P. Greve, C. Luo, B. Wu, J. K. Thompson, Entanglement-enhanced matter-wave interferometry in a high-finesse cavity. Nature 610, 472–477 (2022). 36261551 10.1038/s41586-022-05197-9 PMC9581775

[4] B. Hensen, H. Bernien, A. E. Dréau, A. Reiserer, N. Kalb, M. S. Blok, J. Ruitenberg, R. F. L. Vermeulen, R. N. Schouten, C. Abellán, W. Amaya, V. Pruneri, M. W. Mitchell, M. Markham, D. J. Twitchen, D. Elkouss, S. Wehner, T. H. Taminiau, R. Hanson, Loophole-free Bell inequality violation using electron spins separated by 1.3 kilometres. Nature 526, 682–686 (2015). 26503041 10.1038/nature15759

[5] N. Tomm, A. Javadi, N. O. Antoniadis, D. Najer, M. C. Löbl, A. R. Korsch, R. Schott, S. R. Valentin, A. D. Wieck, A. Ludwig, R. J. Warburton, A bright and fast source of coherent single photons. Nat. Nanotechnol. 16, 399–403 (2021). 33510454 10.1038/s41565-020-00831-x

[6] B. Lubotzky, A. Nazarov, H. Abudayyeh, L. Antoniuk, N. Lettner, V. Agafonov, A. V. Bennett, S. Majumder, V. Chandrasekaran, E. G. Bowes, H. Htoon, J. A. Hollingsworth, A. Kubanek, R. Rapaport, Room-temperature fiber-coupled single-photon sources based on colloidal quantum dots and SiV centers in back-excited nanoantennas. Nano Lett. 24, 640–648 (2024). 38166209 10.1021/acs.nanolett.3c03672 PMC11139382

[7] D. M. Lukin, M. A. Guidry, J. Vučković, Integrated quantum photonics with silicon carbide: Challenges and prospects. PRX Quantum 1, 020102 (2020).

[8] D. P. Ornelas-Huerta, A. N. Craddock, E. A. Goldschmidt, A. J. Hachtel, Y. Wang, P. Bienias, A. V. Gorshkov, S. L. Rolston, J. V. Porto, On-demand indistinguishable single photons from an efficient and pure source based on a Rydberg ensemble. Optica 7, 813–819 (2020).

[9] D. B. Higginbottom, L. Slodička, G. Araneda, L. Lachman, R. Filip, M. Hennrich, R. Blatt, Pure single photons from a trapped atom source. New J. Phys. 18, 093038 (2016).

[10] C. Hamsen, K. N. Tolazzi, T. Wilk, G. Rempe, Two-photon blockade in an atom-driven cavity QED system. Phys. Rev. Lett. 118, 133604 (2017). 28409981 10.1103/PhysRevLett.118.133604

[11] J. Kim, D. Yang, S.-h. Oh, K. An, Coherent single-atom superradiance. Science 359, 662–666 (2018). 29269423 10.1126/science.aar2179

[12] M. Lee, J. Kim, W. Seo, H. G. Hong, Y. Song, R. R. Dasari, K. An, Three-dimensional imaging of cavity vacuum with single atoms localized by a nanohole array. Nat. Comm 5, 3441 (2014). 10.1038/ncomms4441 PMC4073611 24603683

[13] D. Budker, M. Romalis, Optical magnetometry. Nat. Phys. 3, 227–234 (2007).

[14] J. Kitching, Chip-scale atomic devices. Appl. Phys. Rev. 5, 031302 (2018).

[15] R. Zektzer, X. Lu, K. T. Hoang, R. Shrestha, S. Austin, F. Zhou, A. Chanana, G. Holland, D. Westly, P. Lett, A. V. Gorshkov, K. Srinivasan, Strong interactions between integrated microresonators and alkali atomic vapors: Towards single-atom, single-photon operation. Optica 11, 1376–1384 (2024).

[16] R. Shrestha, K. T. Hoang, P. Riley, R. Zektzer, D. Westly, P. Lett, M. T. Hummon, K. Srinivasan, Enabling atom-clad waveguide operation in a microfabricated alkali vapor-photonic integrated circuit. Optica Quantum 4, 62–74 (2026).

[17] L. Stern, B. Desiatov, I. Goykhman, U. Levy, Nanoscale light–matter interactions in atomic cladding waveguides. Nat. Commun. 4, 1548 (2013). 23462991 10.1038/ncomms2554 PMC3615375

[18] J. A. Sedlacek, A. Schwettmann, H. Kübler, R. Löw, T. Pfau, J. P. Shaffer, Microwave electrometry with Rydberg atoms in a vapour cell using bright atomic resonances. Nat. Phys. 8, 819–824 (2012).

[19] K. Levi, A. Giat, L. Golan, E. Talker, L. Stern, Remote chip-scale quantum sensing of magnetic fields. Optica Quantum 3, 84–92 (2025).

[20] V. Gerginov, M. Pomponio, S. Knappe, Scalar magnetometry below 100 fT/Hz^1/2^ in a microfabricated cell. IEEE Sensors J. 20, 12684–12690 (2020). 10.1109/jsen.2020.3002193 PMC9586184 36275194

[21] T. Walker, M. Larsen, Spin-exchange-pumped NMR gyros. Adv. At. Mol. Opt. Phys. 65, 373–401 (2016).

[22] S. Knappe, V. Shah, P. D. D. Schwindt, L. Hollberg, J. Kitching, L. A. Liew, J. Moreland, A microfabricated atomic clock. Appl. Phys. Lett. 85, 1460–1462 (2004).

[23] R. Lutwak, P. Vlitas, M. Varghese, M. Mescher, D. K. Serkland, G. M. Peake, “The miniature atomic clock - Pre-production results,” in *2007 IEEE International Frequency Control Symposium Joint with the 21st European Frequency and Time Forum* (IEEE, 2007), pp. 1327–1333.

[24] C. Li, X. Chai, B. Wei, J. Yang, A. Daruwalla, F. Ayazi, C. Raman, Cascaded collimator for atomic beams traveling in planar silicon devices. Nat. Commun. 10, 1831 (2019). 31015477 10.1038/s41467-019-09647-3 PMC6478944

[25] G. D. Martinez, C. Li, A. Staron, J. Kitching, C. Raman, W. R. McGehee, A chip-scale atomic beam clock. Nat. Commun. 14, 3501 (2023). 37311737 10.1038/s41467-023-39166-1 PMC10264367

[26] N. Jin, C. A. McLemore, D. Mason, J. P. Hendrie, Y. Luo, M. L. Kelleher, P. Kharel, F. Quinlan, S. A. Diddams, P. T. Rakich, Micro-fabricated mirrors with finesse exceeding one million. Optica 9, 965–970 (2022).

[27] H. C. W. Beijerinck, N. F. Verster, Velocity distribution and angular distribution of molecular beams from multichannel arrays. J. Appl. Phys. 46, 2083–2091 (1975).

[28] A. Douahi, L. Nieradko, J. C. Beugnot, J. Dziuban, H. Maillote, S. Guérandel, M. Moraja, C. Gorecki, V. Giordano, Vapour microcell for chip scale atomic frequency standard. Electron. Lett. 43, 279–280 (2007).

[29] K. An, J. J. Childs, R. R. Dasari, M. S. Feld, Microlaser: A laser with one atom in an optical resonator. Phys. Rev. Lett. 73, 3375–3378 (1994). 10057365 10.1103/PhysRevLett.73.3375

[30] C. J. Hood, M. S. Chapman, T. W. Lynn, H. J. Kimble, Real-time cavity QED with single atoms. Phys. Rev. Lett. 80, 4157–4160 (1998).

[31] H. Mabuchi, J. Ye, H. J. Kimble, Full observation of single-atom dynamics in cavity QED. Appl. Phys. B 68, 1095–1108 (1999).

[32] J. A. Sauer, K. M. Fortier, M. S. Chang, C. D. Hamley, M. S. Chapman, Cavity QED with optically transported atoms. Phys. Rev. A 69, 051804 (2004).

[33] M. Khudaverdyan, W. Alt, T. Kampschulte, S. Reick, A. Thobe, A. Widera, D. Meschede, Quantum jumps and spin dynamics of interacting atoms in a strongly coupled atom-cavity system. Phys. Rev. Lett. 103, 123006 (2009). 19792433 10.1103/PhysRevLett.103.123006

[34] M. Mücke, E. Figueroa, J. Bochmann, C. Hahn, K. Murr, S. Ritter, C. J. Villas-Boas, G. Rempe, Electromagnetically induced transparency with single atoms in a cavity. Nature 465, 755–758 (2010). 20463661 10.1038/nature09093

[35] D. Hunger, T. Steinmetz, Y. Colombe, C. Deutsch, T. W. Hänsch, J. Reichel, A fiber Fabry-Perot cavity with high finesse. New J. Phys. 12, 065038 (2010).

[36] M. Uphoff, M. Brekenfeld, G. Rempe, S. Ritter, An integrated quantum repeater at telecom wavelength with single atoms in optical fiber cavities. Appl. Phys. B 122, 46 (2016).

[37] C. A. McLemore, N. Jin, M. L. Kelleher, Y. Luo, D. Lee, Y. Liu, T. Nakamura, D. Mason, P. Rakich, S. A. Diddams, F. Quinlan, Fiber-coupled 2 mL vacuum-gap Fabry–Perot reference cavity for portable laser stabilization. Opt. Lett. 49, 4737–4740 (2024). 39146148 10.1364/OL.531169

[38] Y. Bao, F. Zhou, T. W. LeBrun, J. J. Gorman, Concave silicon micromirrors for stable hemispherical optical microcavities. Opt. Express 25, 15493–15503 (2017). 28788973 10.1364/OE.25.015493 PMC5749248

[39] U. Volz, H. Schmoranzer, Precision lifetime measurements on alkali atoms and on helium by beam-gas-laser spectroscopy. Phys. Scr. T65, 48–56 (1996). 10.1103/PhysRevLett.76.2862 10060810

[40] H. J. Kimble, Strong interactions of single atoms and photons in cavity QED. Phys. Scr. T76, 127–137 (1998).

[41] H. Tanji-Suzuki, W. Chen, R. Landig, J. Simon, V. Vuletić, Interaction between atomic ensembles and optical resonators: Classical description. Adv. At. Mol. Opt. Phys. 60, 201–237 (2011).

[42] Z. Chen, J. G. Bohnet, S. R. Sankar, J. Dai, J. K. Thompson, Conditional spin squeezing of a large ensemble via the vacuum rabi splitting. Phys. Rev. Lett. 106, 133601 (2011). 21517382 10.1103/PhysRevLett.106.133601

[43] I. D. Leroux, M. H. Schleier-Smith, V. Vučković, Implementation of cavity squeezing of a collective atomic spin. Phys. Rev. Lett. 104, 073602 (2010). 20366881 10.1103/PhysRevLett.104.073602

[44] M. H. Schleier-Smith, I. D. Leroux, V. Vučković, States of an ensemble of two-level atoms with reduced quantum uncertainty. Phys. Rev. Lett. 104, 073604 (2010). 20366883 10.1103/PhysRevLett.104.073604

[45] F. Famà, S. Zhou, B. Heizenreder, M. Tang, S. Bennetts, S. B. Jäger, S. A. Schäffer, F. Schreck, Continuous cavity QED with an atomic beam. Phys. Rev. A 110, 063721 (2024).

[46] R. J. Thompson, G. Rempe, H. J. Kimble, Observation of normal-mode splitting for an atom in an optical cavity. Phys. Rev. Lett. 68, 1132–1135 (1992). 10046088 10.1103/PhysRevLett.68.1132

[47] A. N. Nesmeyanov, *Vapor Pressure of the Chemical Elements* (Elsevier, 1963).

[48] J. Gripp, S. L. Mielke, L. A. Orozco, H. J. Carmichael, Anharmonicity of the vacuum Rabi peaks in a many-atom system. Phys. Rev. A 54, R3746–R3749 (1996). 9914028 10.1103/physreva.54.r3746

[49] J. Gea-Banacloche, H. Wu, M. Xiao, Transmission spectrum of Doppler-broadened two-level atoms in a cavity in the strong-coupling regime. Phys. Rev. A 78, 023828 (2008).

[50] R. J. Glauber, The quantum theory of optical coherence. Phys. Rev. 130, 2529–2539 (1963).

[51] G. T. Foster, S. L. Mielke, L. A. Orozco, Intensity correlations in cavity QED. Phys. Rev. A 61, 053821 (2000). 10.1103/PhysRevLett.85.3149 11019288

[52] S. Signorini, L. Pavesi, On-chip heralded single photon sources. AVS Quantum Sci. 2, 041701 (2020).

[53] M. Razavi, I. Söllner, E. Bocquillon, C. Couteau, R. Laflamme, G. Weihs, Characterizing heralded single-photon sources with imperfect measurement devices. J. Phys. B At. Mol. Opt. Phys. 42, 114013 (2009).

[54] H. Carmichael, R. Brecha, P. Rice, Quantum interference and collapse of the wavefunction in cavity QED. Optics Commun. 82, 73–79 (1991).

[55] M. Tavis, F. W. Cummings, Exact solution for an *N*-molecule—Radiation-field Hamiltonian. Phys. Rev. 170, 379–384 (1968).

[56] N. Lambert, E. Giguère, P. Menczel, B. Li, P. Hopf, G. Suárez, M. Gali, J. Lishman, R. Gadhvi, R. Agarwal, A. Galicia, N. Shammah, P. Nation, J. R. Johansson, S. Ahmed, S. Cross, A. Pitchford, F. Nori, QuTiP 5: The quantum toolbox in python. Phys. Rep. 1153, 1–62 (2026).

