# Supplementary material for: A chip-scale atomic beam for nonclassical light
Source: Sci Adv. 2026 Jun 3;12(23):eaec3179. doi: 10.1126/sciadv.aec3179 (PMC13232589; doi:10.1126/sciadv.aec3179)
Supplement: Supplementary file 1 — Supplementary Text Fig. S1 References [file sciadv.aec3179_sm.pdf]

Science Advances
AAAS

Supplementary Materials for

A chip-scale atomic beam for nonclassical light

Braden J. Larsen *et al.*

Corresponding author: Braden J. Larsen, braden.larsen@colorado.edu; James K. Thompson, jkt@jila.colorado.edu

Sci. Adv. **12**, eaec3179 (2026)
DOI: 10.1126/sciadv.aec3179

This PDF file includes:

Supplementary Text
Fig. S1
References

Supplementary Text

Atomic Flux Incident on Mirror Surfaces

A small fraction of the atomic beam is incident on the mirror surface at a highly oblique angle. At 70° C, the total atomic flux of 2×10^{11} atoms/ s results in a flux of approximately 30 atoms/μm^2/s incident on the mirror surface near the cavity mode using the atomic beam model from (*27*). Given that we did not observe any measurable increase in mirror losses from these atoms, it is difficult to estimate what fraction of them are actually deposited onto the mirror surface and the equilibrium density of atoms on the surface.

Aligning the Atomic Beam with the Optical Cavity

To ensure that the angular distribution of the atomic beam is centered with respect to the optical cavity mode, we monitor an additional dispersive feature that appears in the probe transmission spectrum when the atoms have a bias in their velocity class v_z along the cavity axis due to the angular divergence of the atomic beam, as seen in Fig. S1 (*45*). The dispersive feature arises from a modification in the round trip phase of the cavity's circulating field by atoms that experience a simultaneous positive and negative Doppler shift of the cavity field. This phase shift produces a third resonant condition for the cavity, which is only partially attenuated by absorption from the atomic ensemble.

To align the center of the atomic beam with the optical cavity mode, we use a Thorlabs PDR1V translation stage to move the source along the optical cavity axis. When the atomic beam is centered on the cavity axis, this dispersive feature disappears as the cavity field experiences two equal but opposite phase shifts from atoms traveling with equal velocities in opposite directions.

Definitions of $\mathrm{N}_{\mathrm{eff}}$ and g_{eff}

We define an effective atom number $\mathrm{N}_{\mathrm{eff}}$ and effective coupling g_{eff} to account for both the average coupling of the atoms to the cavity and the fluctuations of the average coupling. For N atoms in the atomic beam in a cylindrical volume centered on the cavity axis $V \gg \pi w_0^2 L^2$, the spatial atomic density is just $\rho = N/V$ with Poissonian distributed atom number fluctuations in space. However,

at a specific time t, the atoms generate a collective vacuum Rabi splitting given by:

$$g_{\mathrm{eff}}^2 N_{\mathrm{eff}}(t) = \sum_{i=1}^{N} g_i^2(t) \tag{S1}$$

where we have expressed the time-varying coupling of the atoms to the cavity in terms of a time-independent effective coupling g_{eff} and a time-varying effective atom number $N_{\mathrm{eff}}(t)$.

We now consider the time averaged value $\langle\,\rangle_t$ of the above product and its variance to introduce constraints that define these two quantities. The average can be written as

$$g_{\mathrm{eff}}^2 \langle N_{\mathrm{eff}}(t)\rangle_t = \left\langle \sum_{i=1}^{N} g_i^2(t) \right\rangle_t = \int \rho\, g^2(\mathbf{r})\, dV \tag{S2}$$

where we replace the time average by an ensemble average in the last step.

In the limit of $V \gg \pi w_0^2 L^2$ with ρ held constant, computing the variance of the coupling to the cavity $\mathrm{Var}[g_{\mathrm{eff}}^2 N_{\mathrm{eff}}(t)] = g_{\mathrm{eff}}^4 \langle N_{\mathrm{eff}}^2(t)\rangle_t - g_{\mathrm{eff}}^4 \langle N_{\mathrm{eff}}(t)\rangle_t^2$ leads to the relationship

$$\begin{aligned}\mathrm{Var}[g_{\mathrm{eff}}^2 N_{\mathrm{eff}}(t)] &= \left\langle \sum_{i,j=1}^{N} g_i^2(t)\, g_j^2(t) \right\rangle_t - \left\langle \sum_{i=1}^{N} g_i^2(t) \right\rangle_t^2 \\ &= \int \rho\, g^4(\mathbf{r})\, dV\end{aligned}$$

where we have assumed no correlations in the fluctuations in coupling of atom i and j, and again replace the time average by an ensemble average.

We now impose the final constraint that the fluctuations in the effective atom number are Poissonian such that

$$\mathrm{Var}[N_{\mathrm{eff}}(t)] = \langle N_{\mathrm{eff}}(t)\rangle_t \tag{S3}$$

from which we find that the effective coupling g_{eff} is given by

$$
\begin{aligned}
g_{\text{eff}} &= \sqrt{\frac{\int_0^L \int_0^\infty \rho g^4(\mathbf{r}) r dr dz}{\int_0^L \int_0^\infty \rho g^2(\mathbf{r}) r dr dz}} \\
&= g_0 \sqrt{\frac{\int_0^L \int_0^\infty \cos^4(2\pi z/\lambda_a) e^{-4r^2/w_0^2} r dr dz}{\int_0^L \int_0^\infty \cos^2(2\pi z/\lambda_a) e^{-2r^2/w_0^2} r dr dz}} \\
&= g_0 \sqrt{\frac{\int_0^\infty e^{-4r^2/w_0^2} r dr}{\int_0^\infty e^{-2r^2/w_0^2} r dr}} \sqrt{\frac{\int_0^L \cos^4(2\pi z/\lambda_a) dz}{\int_0^L \cos^2(2\pi z/\lambda_a) dz}} = \frac{1}{\sqrt{2}} \frac{\sqrt{3}}{2} g_0 = \sqrt{\frac{3}{8}} g_0 \\
&= 2\pi \times 16(1) \text{ MHz}
\end{aligned}
\tag{S4}
$$

The factor of $\sqrt{3/8}$ can be thought of as a product of the contributions from the radial and axial components of the inhomogeneity, which are $1/\sqrt{2}$ and $\sqrt{3}/2$ respectively. This means, for example, that an atomic beam passing through only antinodes of the cavity mode would just have $g_{\text{eff}} = g/\sqrt{2}$. The effective atom number $N_{\text{eff}}(t)$ is given by

$$
N_{\text{eff}}(t) = \frac{8}{3} \sum_i \frac{g_i^2(t)}{g_0^2} \tag{S5}
$$

where the sum is, in principle, taken over all atoms i within some large volume V in the limit $V \longrightarrow \infty$. The contribution of atoms to the collective coupling rapidly decays beyond a distance w_0 away from the cavity axis, and the sum converges quickly to a finite value for $V \gg \pi w_0^2 L^2$. By converting the ensemble average of this expression into a time average, we can write the time-averaged effective atom number $N_{\text{eff}} \equiv \langle N_{\text{eff}}(t) \rangle_t$ in terms of the atom number density ρ:

$$
N_{\text{eff}} = \frac{2}{3} \pi w_0^2 L \rho \tag{S6}
$$

This specifies an effective volume $V_{\text{eff}} = \frac{2}{3} \pi w_0^2 L$, within which the mean number of atoms ρV_{eff} is equal to N_{eff}. For a flux F of atoms entering the cavity at mean velocity v, we can write $\rho = \frac{F}{v}$ and thus

$$
N_{\text{eff}} = V_{\text{eff}} \frac{F}{v} \tag{S7}
$$

Since ρ and F are not easily measurable quantities in our system, N_{eff} can be determined in practice from the vacuum Rabi splitting or estimated from $g^{(2)}(\tau)$.

Modeling the Bunched Envelope of $g^{(2)}(\tau)$

The bunched envelope featured in the calculated $g^{(2)}(\tau)$ disagrees with the expectation that $g^{(2)}(0) < 1$ for a single atom trapped in the cavity mode at all times. However, our atom number fluctuates as atoms enter the cavity mode at random times, and the rate of photons emitted from the cavity fluctuates in turn (*51, 54*). Consider a simple model where the atoms are independent linear scatterers, which scatter photons from the excitation laser at a total rate Γ_{tot} proportional to N_{eff}. In this model, we would expect:

$$g^{(2)}(0) = \frac{\langle \Gamma_{\text{tot}}^2 \rangle}{\langle \Gamma_{\text{tot}} \rangle^2} = 1 + \frac{\text{Var}(N_{\text{eff}})}{(N_{\text{eff}})^2} = 1 + \frac{1}{N_{\text{eff}}} > 1 \tag{S8}$$

where we used the fact that N_{eff} is defined using equation S3. This means that $g^{(2)}(0) > 1$, which looks like photon *bunching*. Note that $g^{(2)}(0)$ is larger at smaller atom number.

This picture is complicated in reality by non-classical correlations between the emitted photons, as well as the fact that the rate of emitted photons is only exactly proportional to N_{eff} in the limit of small coupling g. Even with these complications, however, the same argument still approximately holds for our system. This phenomenon is responsible for the bunched envelope in $g^{(2)}(\tau)$.

Theoretical Model and Monte-Carlo Wavefunction Simulations

The Hamiltonian describing a total of N_t atoms transiting the cavity will be approximated by a two-level atomic Hamiltonian with transition frequency ω_a, and an excitation laser at frequency ω_d and Rabi frequency $\Omega_i(t)$ as

$$\hat{H}_a = \frac{1}{2} \sum_{i=1}^{N_t} \left(\omega_z \hat{\sigma}_i^z + \frac{1}{2} \left(\Omega_i(t) \hat{\sigma}_i^+ + \Omega_i^*(t) \hat{\sigma}_i^- \right) \right) \tag{S9}$$

where $\hat{\sigma}_i^\alpha$ is the usual Pauli spin operator for the ith atom with $\alpha = \{x, y, z, +, -\}$.

The cavity is described by a quantum harmonic oscillator Hamiltonian

$$\hat{H}_c = \omega_c \left(\hat{c}^\dagger \hat{c} + \frac{1}{2} \right), \tag{S10}$$

where $\hat{c}$ and $\hat{c}^\dagger$ are bosonic creation and annihilation operators for the cavity field.

For non-classical light generation the cavity and drive are both tuned to resonance with the atomic transition frequency so that $\omega_c = \omega_d = \omega_a$. Moving into a rotating frame at ω_a, and including the Tavis-Cummings atom-cavity interaction (*55*), we have a total Hamiltonian

$$\hat{H}_t = \sum_{i=1}^{N_t} \left[g_i(t) \left(\hat{\sigma}_i^+ \hat{c} + \hat{\sigma}_i^- \hat{c}^\dagger \right) + \frac{1}{2} \left(\Omega_i(t) \hat{\sigma}_i^+ + \Omega_i^*(t) \hat{\sigma}_i^- \right) \right] \tag{S11}$$

where the time-varying components are

$$g_i(t) = g_0 e^{-\frac{x_i^2(t)+y_i^2(t)}{w_0^2}} \cos\left(\frac{2\pi z_i(t)}{\lambda_a} \right) \tag{S12}$$

and

$$\Omega_i(t) = \Omega e^{2\pi \imath y_i(t)/\lambda_a} e^{-\frac{(x_i(t)^2+z_i(t)^2)}{w_d^2}} \tag{S13}$$

which represent the coupling of the ith atom to the standing wave cavity mode and excitation laser, respectively. w_d is the waist of the drive beam at the cavity mode, taken to be 40 μm. The atoms are approximated as following classical ballistic trajectories $\mathbf{r}_i(t) = x_i(t)\hat{x} + y_i(t)\hat{y} + z_i(t)\hat{z}$. Cavity decay at rate κ and single-atom decay Γ from the excited state via spontaneous emission into free space are described by jump operators $\sqrt{\kappa}c$ and $\sqrt{\Gamma}\sigma_i^-$, respectively.

Monte-Carlo Wavefunction (MCWF) simulations are performed using QuTiP (*56*) and compared to data in Fig. 3 and 4 of the main text. We use a truncated Hilbert space that allows for a maximum of two atoms and five photons in the cavity at once. The parameters of the system are such that it is very rare for these limits to be exceeded.

The simulated atomic beam flux is chosen to match the amplitude of the bunched envelope, and the parameters $g_0 = 2\pi \times 15$ MHz and $\Omega = 2\pi \times 11$ MHz for a two-level atom are adjusted in the simulation to more accurately describe the central dip. These values should be compared with the predicted values for a multi-level atom $g_0 = 2\pi \times 22(2)$ MHz and $\Omega = 2\pi \times 18^{+2}_{-9}$ MHz in a simple model that averages over Clebsch-Gordan coefficients.

The classical ballistic trajectories $\mathbf{r}_i(t)$ for each atom is randomly sampled from a distribution of velocities, angles, positions and arrival times that is representative of our thermal beam geometry. The atomic trajectories are generated so that atoms arrive at the cavity with a rate consistent with $N_{\text{eff}} \approx 0.1$. Atoms are added or removed from the simulation when they enter or exit the y-z plane at $3w_0$ from the center of the cavity mode, where the coupling g_i is roughly 0.25% of its maximum

possible value g_0. Each atom is assigned an initial position z_{0i} along the cavity axis between 0 and λ_a and an initial height y_{0i} in the excitation laser between $-w_0$ and w_0. The velocity v of each atom is selected from the Maxwell-Boltzmann distribution for the atomic beam flux $F(v)$, which is given by:

$$F(v) = \frac{1}{2v_{rms}} \left(\frac{v}{v_{rms}}\right)^3 e^{-\frac{v^2}{2v_{rms}^2}} \tag{S14}$$

where $v_{rms} = \sqrt{k_B T/m_{\mathrm{Rb}}}$ is the rms velocity of the rubidium beam, T is the temperature of the atomic beam, and m_{Rb} is the atomic mass of ^{87}Rb. The angular divergence of the beam is represented by a random sampling of each atom's angle from uniform distributions along the $\hat{y}$ and $\hat{z}$ directions. The distribution along the $\hat{y}$ direction takes on the full angular divergence of atomic beam $\theta_{y_{rms}} = 43$ mrad, while along $\hat{z}$ it is restricted to $\theta_{z_{rms}} = 8$ mrad. The trajectories are propagated through time as $\mathbf{r}_i(t) = (-3w_0 + v_{xi}t)\hat{x} + (y_{0i} + v_{yi}t)\hat{y} + (z_{0i} + v_{zi}t)\hat{z}$.

A photon click is recorded whenever the cavity decay jump operator is applied. The generated record of time-stamped photon clicks is then supplied to our data analysis routine to generate a desired photon correlation function such as $g^{(2)}(\tau)$ or $g^{(3)}(\tau_1, \tau_2)$.

Averaging Heralded Correlation Measurements

When measuring a heralded $g_h^{(2)}(\tau_a, \tau_b)$ function, we store the photon counts from all three detectors without assigning any one detector to be the herald. The choice of herald detector is arbitrary, and can be made later in post-processing. We can also arbitrarily choose which of the two remaining detectors is labeled as detector a and which is labeled as detector b. In total, there are six possible permutations of detector labels (a, b, c), and $g_h^{(2)}(\tau_a, \tau_b)$ can be calculated for any of these permutations. All possible permutations should produce the same $g_h^{(2)}(\tau_a, \tau_b)$ up to random shot noise, so we average over all permutations in (Fig. 4) to reduce this noise. This introduces redundancies in the data by forcing symmetries to hold exactly (e.g. $g_h^{(2)}(\tau_a, \tau_b) = g_h^{(2)}(\tau_b, \tau_a)$), but effectively increases the signal-to-noise ratio by allowing any detector to be the herald.

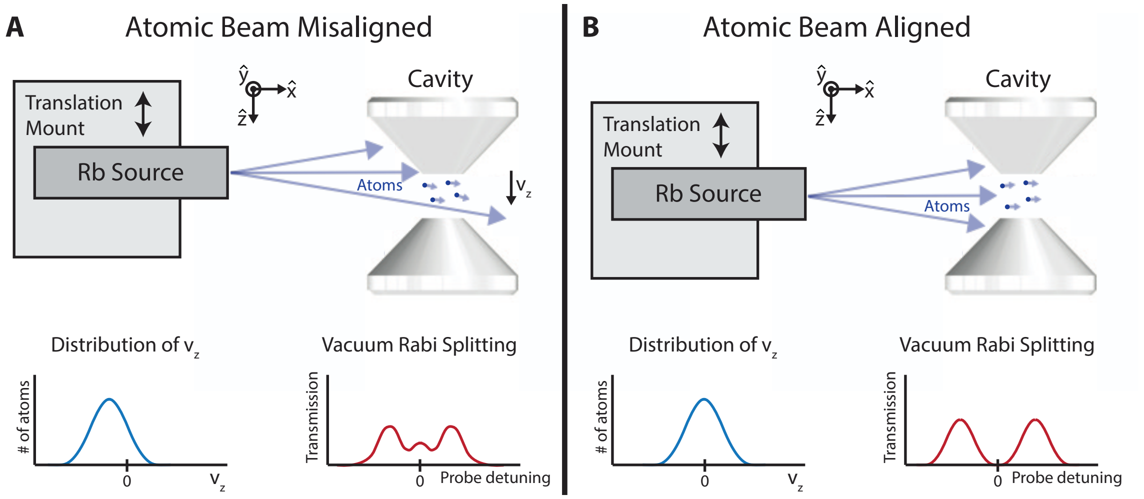

Figure S1: **Procedure for aligning the atomic beam. (A)** When the atomic beam is misaligned, transiting atoms have a bias in their velocity v_z along the cavity axis. This results in a third peak appearing in the VRS probe transmission spectrum. **(B)** When this misalignment is corrected using the translation stage, this third peak disappears and we know that the beam is aligned. All plots are for conceptual purposes and not real data.

REFERENCES

1. M. Zou, Y. M. He, Y. Huang, J. Y. Zhao, B. C. Li, Y. P. Guo, X. Ding, M. C. Xu, R. Z. Liu, G. Y. Zou, Z. Ning, X. You, H. Wang, W. X. Pan, H. T. Zhu, M. Y. Zheng, X. P. Xie, D. Qin, X. Jiang, Y. H. Huo, Q. Zhang, C. Y. Lu, X. Ma, T. Y. Chen, J. W. Pan, Realization of an untrusted intermediate relay architecture using a quantum dot single-photon source. *Nat. Phys.* **21**, 1670–1677 (2025).

2. J. M. Arrazola, V. Bergholm, K. Brádler, T. R. Bromley, M. J. Collins, I. Dhand, A. Fumagalli, T. Gerrits, A. Goussev, L. G. Helt, J. Hundal, T. Isacsson, R. B. Israel, J. Izaac, S. Jahangiri, R. Janik, N. Killoran, S. P. Kumar, J. Lavoie, A. E. Lita, D. H. Mahler, M. Menotti, B. Morrison, S. W. Nam, L. Neuhaus, H. Y. Qi, N. Quesada, A. Repingon, K. K. Sabapathy, M. Schuld, D. Su, J. Swinarton, A. Száva, K. Tan, P. Tan, V. D. Vaidya, Z. Vernon, Z. Zabaneh, Y. Zhang, Quantum circuits with many photons on a programmable nanophotonic chip. *Nature* **591**, 54–60 (2021).

3. G. P. Greve, C. Luo, B. Wu, J. K. Thompson, Entanglement-enhanced matter-wave interferometry in a high-finesse cavity. *Nature* **610**, 472–477 (2022).

4. B. Hensen, H. Bernien, A. E. Dréau, A. Reiserer, N. Kalb, M. S. Blok, J. Ruitenberg, R. F. L. Vermeulen, R. N. Schouten, C. Abellán, W. Amaya, V. Pruneri, M. W. Mitchell, M. Markham, D. J. Twitchen, D. Elkouss, S. Wehner, T. H. Taminiau, R. Hanson, Loophole-free Bell inequality violation using electron spins separated by 1.3 kilometres. *Nature* **526**, 682–686 (2015).

5. N. Tomm, A. Javadi, N. O. Antoniadis, D. Najer, M. C. Löbl, A. R. Korsch, R. Schott, S. R. Valentin, A. D. Wieck, A. Ludwig, R. J. Warburton, A bright and fast source of coherent single photons. *Nat. Nanotechnol.* **16**, 399–403 (2021).

6. B. Lubotzky, A. Nazarov, H. Abudayyeh, L. Antoniuk, N. Lettner, V. Agafonov, A. V. Bennett, S. Majumder, V. Chandrasekaran, E. G. Bowes, H. Htoon, J. A. Hollingsworth, A. Kubanek, R. Rapaport, Room-temperature fiber-coupled single-photon sources based on colloidal quantum dots and SiV centers in back-excited nanoantennas. *Nano Lett.* **24**, 640–648 (2024).

7. D. M. Lukin, M. A. Guidry, J. Vučković, Integrated quantum photonics with silicon carbide: Challenges and prospects. *PRX Quantum* **1**, 020102 (2020).

8. D. P. Ornelas-Huerta, A. N. Craddock, E. A. Goldschmidt, A. J. Hachtel, Y. Wang, P. Bienias, A. V. Gorshkov, S. L. Rolston, J. V. Porto, On-demand indistinguishable single photons from an efficient and pure source based on a Rydberg ensemble. *Optica* **7**, 813–819 (2020).

9. D. B. Higginbottom, L. Slodička, G. Araneda, L. Lachman, R. Filip, M. Hennrich, R. Blatt, Pure single photons from a trapped atom source. *New J. Phys.* **18**, 093038 (2016).

10. C. Hamsen, K. N. Tolazzi, T. Wilk, G. Rempe, Two-photon blockade in an atom-driven cavity QED system. *Phys. Rev. Lett.* **118**, 133604 (2017).

11. J. Kim, D. Yang, S.-h. Oh, K. An, Coherent single-atom superradiance. *Science* **359**, 662–666 (2018).

12. M. Lee, J. Kim, W. Seo, H. G. Hong, Y. Song, R. R. Dasari, K. An, Three-dimensional imaging of cavity vacuum with single atoms localized by a nanohole array. *Nat. Comm* **5**, 3441 (2014).

13. D. Budker, M. Romalis, Optical magnetometry. *Nat. Phys.* **3**, 227–234 (2007).

14. J. Kitching, Chip-scale atomic devices. *Appl. Phys. Rev.* **5**, 031302 (2018).

15. R. Zektzer, X. Lu, K. T. Hoang, R. Shrestha, S. Austin, F. Zhou, A. Chanana, G. Holland, D. Westly, P. Lett, A. V. Gorshkov, K. Srinivasan, Strong interactions between integrated microresonators and alkali atomic vapors: Towards single-atom, single-photon operation. *Optica* **11**, 1376–1384 (2024).

16. R. Shrestha, K. T. Hoang, P. Riley, R. Zektzer, D. Westly, P. Lett, M. T. Hummon, K. Srinivasan, Enabling atom-clad waveguide operation in a microfabricated alkali vapor-photonic integrated circuit. *Optica Quantum* **4**, 62–74 (2026).

17. L. Stern, B. Desiatov, I. Goykhman, U. Levy, Nanoscale light–matter interactions in atomic cladding waveguides. *Nat. Commun.* **4**, 1548 (2013).

18. J. A. Sedlacek, A. Schwettmann, H. Kübler, R. Löw, T. Pfau, J. P. Shaffer, Microwave electrometry with Rydberg atoms in a vapour cell using bright atomic resonances. *Nat. Phys.* **8**, 819–824 (2012).

19. K. Levi, A. Giat, L. Golan, E. Talker, L. Stern, Remote chip-scale quantum sensing of magnetic fields. *Optica Quantum* **3**, 84–92 (2025).

20. V. Gerginov, M. Pomponio, S. Knappe, Scalar magnetometry below 100 fT/Hz$^{1/2}$ in a microfabricated cell. *IEEE Sensors J.* **20**, 12684–12690 (2020).

21. T. Walker, M. Larsen, Spin-exchange-pumped NMR gyros. *Adv. At. Mol. Opt. Phys.* **65**, 373–401 (2016).

22. S. Knappe, V. Shah, P. D. D. Schwindt, L. Hollberg, J. Kitching, L. A. Liew, J. Moreland, A microfabricated atomic clock. *Appl. Phys. Lett.* **85**, 1460–1462 (2004).

23. R. Lutwak, P. Vlitas, M. Varghese, M. Mescher, D. K. Serkland, G. M. Peake, "The miniature atomic clock - Pre-production results," in *2007 IEEE International Frequency Control Symposium Joint with the 21st European Frequency and Time Forum* (IEEE, 2007), pp. 1327–1333.

24. C. Li, X. Chai, B. Wei, J. Yang, A. Daruwalla, F. Ayazi, C. Raman, Cascaded collimator for atomic beams traveling in planar silicon devices. *Nat. Commun.* **10**, 1831 (2019).

25. G. D. Martinez, C. Li, A. Staron, J. Kitching, C. Raman, W. R. McGehee, A chip-scale atomic beam clock. *Nat. Commun.* **14**, 3501 (2023).

26. N. Jin, C. A. McLemore, D. Mason, J. P. Hendrie, Y. Luo, M. L. Kelleher, P. Kharel, F. Quinlan, S. A. Diddams, P. T. Rakich, Micro-fabricated mirrors with finesse exceeding one million. *Optica* **9**, 965–970 (2022).

27. H. C. W. Beijerinck, N. F. Verster, Velocity distribution and angular distribution of molecular beams from multichannel arrays. *J. Appl. Phys.* **46**, 2083–2091 (1975).

28. A. Douahi, L. Nieradko, J. C. Beugnot, J. Dziuban, H. Maillote, S. Guérandel, M. Moraja, C. Gorecki, V. Giordano, Vapour microcell for chip scale atomic frequency standard. *Electron. Lett.* **43**, 279–280 (2007).

29. K. An, J. J. Childs, R. R. Dasari, M. S. Feld, Microlaser: A laser with one atom in an optical resonator. *Phys. Rev. Lett.* **73**, 3375–3378 (1994).

30. C. J. Hood, M. S. Chapman, T. W. Lynn, H. J. Kimble, Real-time cavity QED with single atoms. *Phys. Rev. Lett.* **80**, 4157–4160 (1998).

31. H. Mabuchi, J. Ye, H. J. Kimble, Full observation of single-atom dynamics in cavity QED. *Appl. Phys. B* **68**, 1095–1108 (1999).

32. J. A. Sauer, K. M. Fortier, M. S. Chang, C. D. Hamley, M. S. Chapman, Cavity QED with optically transported atoms. *Phys. Rev. A* **69**, 051804 (2004).

33. M. Khudaverdyan, W. Alt, T. Kampschulte, S. Reick, A. Thobe, A. Widera, D. Meschede, Quantum jumps and spin dynamics of interacting atoms in a strongly coupled atom-cavity system. *Phys. Rev. Lett.* **103**, 123006 (2009).

34. M. Mücke, E. Figueroa, J. Bochmann, C. Hahn, K. Murr, S. Ritter, C. J. Villas-Boas, G. Rempe, Electromagnetically induced transparency with single atoms in a cavity. *Nature* **465**, 755–758 (2010).

35. D. Hunger, T. Steinmetz, Y. Colombe, C. Deutsch, T. W. Hänsch, J. Reichel, A fiber Fabry-Perot cavity with high finesse. *New J. Phys.* **12**, 065038 (2010).

36. M. Uphoff, M. Brekenfeld, G. Rempe, S. Ritter, An integrated quantum repeater at telecom wavelength with single atoms in optical fiber cavities. *Appl. Phys. B* **122**, 46 (2016).

37. C. A. McLemore, N. Jin, M. L. Kelleher, Y. Luo, D. Lee, Y. Liu, T. Nakamura, D. Mason, P. Rakich, S. A. Diddams, F. Quinlan, Fiber-coupled 2 mL vacuum-gap Fabry–Perot reference cavity for portable laser stabilization. *Opt. Lett.* **49**, 4737–4740 (2024).

38. Y. Bao, F. Zhou, T. W. LeBrun, J. J. Gorman, Concave silicon micromirrors for stable hemispherical optical microcavities. *Opt. Express* **25**, 15493–15503 (2017).

39. U. Volz, H. Schmoranzer, Precision lifetime measurements on alkali atoms and on helium by beam-gas-laser spectroscopy. *Phys. Scr.* **T65**, 48–56 (1996).

40. H. J. Kimble, Strong interactions of single atoms and photons in cavity QED. *Phys. Scr.* **T76**, 127–137 (1998).

41. H. Tanji-Suzuki, W. Chen, R. Landig, J. Simon, V. Vuletić, Interaction between atomic ensembles and optical resonators: Classical description. *Adv. At. Mol. Opt. Phys.* **60**, 201–237 (2011).

42. Z. Chen, J. G. Bohnet, S. R. Sankar, J. Dai, J. K. Thompson, Conditional spin squeezing of a large ensemble via the vacuum rabi splitting. *Phys. Rev. Lett.* **106**, 133601 (2011).

43. I. D. Leroux, M. H. Schleier-Smith, V. Vučković, Implementation of cavity squeezing of a collective atomic spin. *Phys. Rev. Lett.* **104**, 073602 (2010).

44. M. H. Schleier-Smith, I. D. Leroux, V. Vučković, States of an ensemble of two-level atoms with reduced quantum uncertainty. *Phys. Rev. Lett.* **104**, 073604 (2010).

45. F. Famà, S. Zhou, B. Heizenreder, M. Tang, S. Bennetts, S. B. Jäger, S. A. Schäffer, F. Schreck, Continuous cavity QED with an atomic beam. *Phys. Rev. A* **110**, 063721 (2024).

46. R. J. Thompson, G. Rempe, H. J. Kimble, Observation of normal-mode splitting for an atom in an optical cavity. *Phys. Rev. Lett.* **68**, 1132–1135 (1992).

47. A. N. Nesmeyanov, *Vapor Pressure of the Chemical Elements* (Elsevier, 1963).

48. J. Gripp, S. L. Mielke, L. A. Orozco, H. J. Carmichael, Anharmonicity of the vacuum Rabi peaks in a many-atom system. *Phys. Rev. A* **54**, R3746–R3749 (1996).

49. J. Gea-Banacloche, H. Wu, M. Xiao, Transmission spectrum of Doppler-broadened two-level atoms in a cavity in the strong-coupling regime. *Phys. Rev. A* **78**, 023828 (2008).

50. R. J. Glauber, The quantum theory of optical coherence. *Phys. Rev.* **130**, 2529–2539 (1963).

51. G. T. Foster, S. L. Mielke, L. A. Orozco, Intensity correlations in cavity QED. *Phys. Rev. A* **61**, 053821 (2000).

52. S. Signorini, L. Pavesi, On-chip heralded single photon sources. *AVS Quantum Sci.* **2**, 041701 (2020).

53. M. Razavi, I. Söllner, E. Bocquillon, C. Couteau, R. Laflamme, G. Weihs, Characterizing heralded single-photon sources with imperfect measurement devices. *J. Phys. B At. Mol. Opt. Phys.* **42**, 114013 (2009).

54. H. Carmichael, R. Brecha, P. Rice, Quantum interference and collapse of the wavefunction in cavity QED. *Optics Commun.* **82**, 73–79 (1991).

55. M. Tavis, F. W. Cummings, Exact solution for an *N*-molecule—Radiation-field Hamiltonian. *Phys. Rev.* **170**, 379–384 (1968).

56. N. Lambert, E. Giguère, P. Menczel, B. Li, P. Hopf, G. Suárez, M. Gali, J. Lishman, R. Gadhvi, R. Agarwal, A. Galicia, N. Shammah, P. Nation, J. R. Johansson, S. Ahmed, S. Cross, A. Pitchford, F. Nori, QuTiP 5: The quantum toolbox in python. *Phys. Rep.* **1153**, 1–62 (2026).
